# Time to surgery following chronic subdural hematoma: post hoc analysis of a prospective cohort study

**DOI:** 10.1136/bmjsit-2019-000012

**Published:** 2019-12-16

**Authors:** Sara Venturini, Daniel M Fountain, Laurence J Glancz, Laurent J Livermore, Ian C Coulter, Simon Bond, Basil Matta, Thomas Santarius, Peter J Hutchinson, Paul M Brennan, Angelos G Kolias, The British Neurosurgical Trainee Research Collaborative (BNTRC), FT Afshari

**Affiliations:** 1 Department of Neurosurgery, Aberdeen Royal Infirmary, Aberdeen, UK; 2 Division of Neurosurgery, Department of Clinical Neurosciences, University of Cambridge & Addenbrooke's Hospital, Cambridge, UK; 3 Department of Neurosurgery, Queen's Medical Centre, Nottingham, UK; 4 Department of Neurosurgery, John Radcliffe Hospital, Oxford, UK; 5 Department of Neurosurgery, Royal Victoria Infirmary, Newcastle upon Tyne, UK; 6 Cambridge Clinical Trials Unit, Cambridge University Hospitals NHS Foundation Trust, Cambridge, UK; 7 Department of Anaesthesia, Addenbrooke's Hospital, Cambridge, Cambridgeshire, UK; 8 Translational Neurosurgery, Centre for Clinical Brain Sciences, The University of Edinburgh, Edinburgh, UK

**Keywords:** cohort study, outcomes research, real world evidence

## Abstract

**Background:**

Chronic subdural hematoma (CSDH) is a common neurological condition; surgical evacuation is the mainstay of treatment for symptomatic patients. No clear evidence exists regarding the impact of timing of surgery on outcomes. We investigated factors influencing time to surgery and its impact on outcomes of interest.

**Methods:**

Patients with CSDH who underwent burr-hole craniostomy were included. This is a subset of data from a prospective observational study conducted in the UK. Logistic mixed modelling was performed to examine the factors influencing time to surgery. The impact of time to surgery on discharge modified Rankin Scale (mRS), complications, recurrence, length of stay and survival was investigated with multivariable logistic regression analysis.

**Results:**

656 patients were included. Time to surgery ranged from 0 to 44 days (median 1, IQR 1–3). Older age, more favorable mRS on admission, high preoperative Glasgow Coma Scale score, use of antiplatelet medications, comorbidities and bilateral hematomas were associated with increased time to surgery. Time to surgery showed a significant positive association with length of stay; it was not associated with outcome, complication rate, reoperation rate, or survival on multivariable analysis. There was a trend for patients with time to surgery of ≥7 days to have lower odds of favorable outcome at discharge (p=0.061).

**Conclusions:**

This study provides evidence that time to surgery does not substantially impact on outcomes following CSDH. However, increasing time to surgery is associated with increasing length of stay. These results should not encourage delaying operations for patients when they are clinically indicated.

**Key messages**

**What is already known about this subject?**

- Chronic subdural hematoma is a common condition.
- Surgical evacuation of the hematoma is often the treatment.
- Elderly patients are commonly affected.

**What are the new findings?**

- Factors associated with longer time to surgery were older age, antiplatelet medication use, low modified Rankin Scale on admission.
- There is a positive association between time to surgery and length of stay.

**How might these results affect future research or surgical practice?**

- Our results emphasize the need to base time to surgery decisions on clinical assessment. Further research is required to explore this topic fully.

## Introduction

Chronic subdural hematoma (CSDH) is a common neurosurgical condition, resulting from the formation of liquefied blood in the subdural space between the dura and arachnoid mater, usually following trivial trauma. Occurring in up to 14 per 100 000 people per year, the mainstay of treatment for symptomatic CSDH is surgical evacuation. Patients with CSDH are typically elderly, with a high incidence of comorbidities and iatrogenic coagulopathy and thrombopathy.^1^ Often, there are practical reasons for delaying surgery in symptomatic patients; for example, the British Society of Haematology recommends delaying surgery until 24 hours after the last dose of direct oral anticoagulants, extended to 48 hours if the patient has renal impairment or is on a direct thrombin inhibitor.^2^ For other patients, delays may result from non-evidence-based decision-making and logistical issues within health services.

The timing of neurosurgical intervention in CSDH is relatively understudied, with no high-quality evidence regarding the optimal time to intervene. Studies of patients with acute traumatic extra-axial hematomas have previously demonstrated an improved outcome with surgical intervention within 4 hours of injury but these findings cannot be directly translated to CSDH, as the pathophysiology and clinical presentation of the latter differs significantly from the former.^3 4^ Additionally, more recent studies have challenged the ‘four hours’ rule.^5 6^ Most recently, a large UK study of patients with acute subdural hematoma found no evidence of an effect of time interval from injury to craniotomy (n=2498 patients, p=0.4203).^7^

The management of CSDH was recently investigated through a prospective, multicenter, observational cohort study in the UK.^8 9^ Variables associated with a favorable outcome included lower age, better initial modified Rankin Scale (mRS) score, higher preoperative Glasgow Coma Scale (GCS) score, using more than one burr-hole, inserting a subdural drain and avoiding prescribed bed rest. In the context of the outstanding research questions regarding timing of surgery and the above findings, this post hoc analysis aims to investigate (1) factors associated with time to surgery and (2) the impact of time to surgery on functional outcome, complications, recurrence, length of stay, and survival.

## Methods

Data were collected prospectively as described in the primary study publication and protocol.^8 9^ The main study protocol was approved by the Academic Committee of the Society of British Neurological Surgeons (SBNS). The study was supported by the SBNS and formed part of the Neurosurgical National Audit Program. Local governance approvals were in place in each participating neurosurgical unit. Data for 1205 patients referred to 26 of the 33 UK and Ireland neurosurgical units were prospectively collected between May 2013 and January 2014. This paper examined a subset of this sample, excluding patients (1) not transferred to a neurosurgical unit, (2) with a previous history of ipsilateral CSDH, (3) who did not have burr-hole evacuation, (4) with unavailable time to surgery data and (5) with a cerebrospinal fluid shunt. This paper is reported in accordance with the Strengthening the Reporting of Observational Studies in Epidemiology statement for cohort studies.^10^

Time from neurosurgical referral to surgery was categorized as follows: 0, 1, 2, 3–6, and 7 or more days. The time interval was calculated by subtracting the date of referral from date of operation (specific times in hours were not available). For example, a patient referred on 1 December and operated on 1, would be categorized as ‘time to surgery 0 days’. A patient referred on 1 December and operated on the 2 would be categorized as ‘time to surgery 1 day’. Univariable categorical comparisons on pair-wise data were undertaken using Fisher’s exact testing. Comparisons of continuous data were undertaken using Kruskal-Wallis testing.

We performed mixed modelling as a logistic model to examine factors influencing time to surgery. In this model, time to surgery was the dependent variable, with a cut-off for early surgical intervention based on the median of data collected and data distribution. Independent variables were selected based on clinical relevance and included if found to be significant (p<0.05) with concomitant improvement in the model, as demonstrated by the Akaike Information Criterion (AIC) under multivariable analysis.^11^ The neurosurgical center was included as a random effect due to hypothesized differences in median time to surgery and heterogeneous sampling between centers.

Time to surgery was modelled as a categorical, independent variable to evaluate its impact on (1) discharge mRS, expressed as either favorable (0–3) or unfavorable (4–6), (2) complications, which included surgical site infection, seizures, new deficits, respiratory tract infections, arrhythmias, venous thromboembolism, myocardial infarctions and cerebrovascular accidents, (3) symptomatic recurrence requiring repeat surgery, (4) length of stay, and (5) survival. Time to surgery was categorized as described above. Each time interval category was compared with 0 days for the calculation of ORs. Covariates were selected based on the results of the logistic mixed modelling and in accordance with previously published models of these data, including patient age as a continuous variable, initial mRS and preoperative GCS as ordinal variables, and antiplatelet medication, clot density, 2 or more burr-holes, bilateral operation, drain insertion, management with bed rest and provision of high-flow oxygen as dichotomous variables. Results were not repeated if consistent with previously published material.^8^

Logistic mixed modelling and regression analyses were performed using *rms* and *lme4* packages in R v 3.2.1.^12 13^ A single p value was obtained by computing a Wald χ^2^ pooled statistic of all coefficients of the variable of interest and was significant if p<0.05. All descriptive graphics were completed using the ggplot2 package in R 3.2.1.^14^

## Results

**Figure 1:**
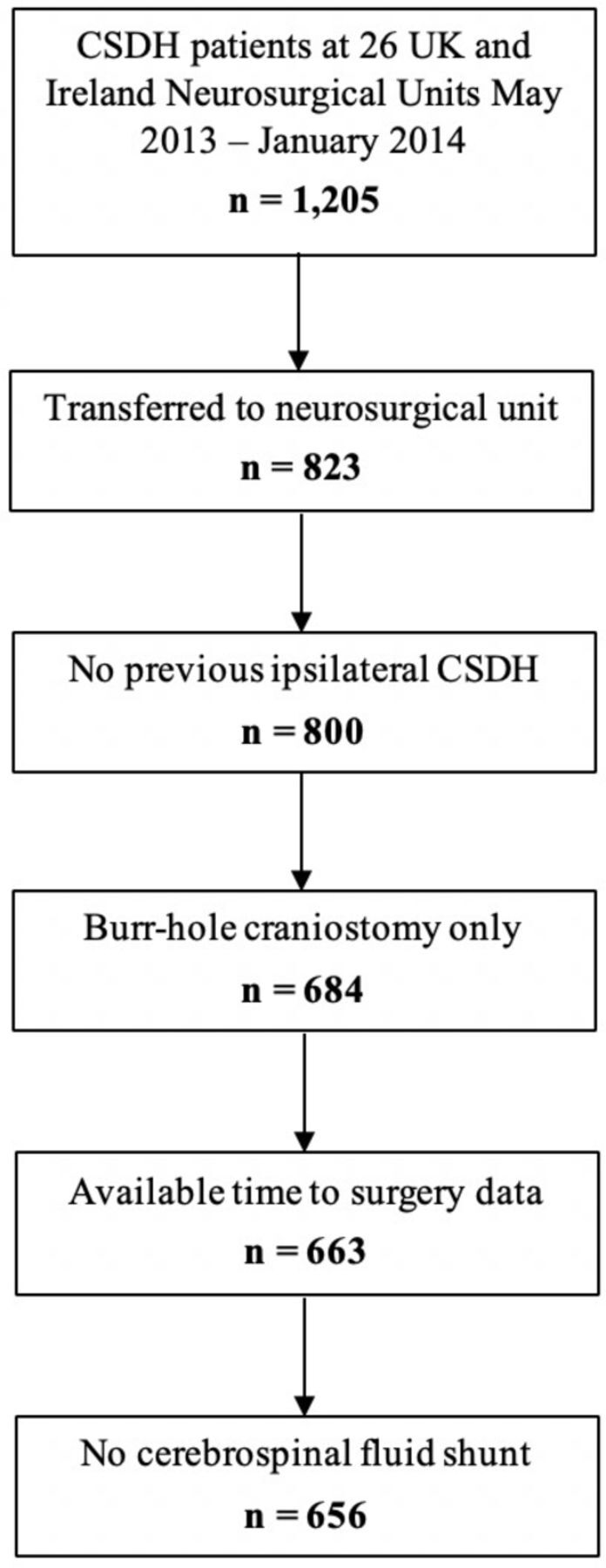
Flow diagram of included patients. CSDH, chronic subdural hematoma.

Of the 1205 patients examined, 656 patients met the inclusion criteria for this study. These patients all had a primary unilateral or bilateral CSDH (in the absence of a shunt) drained via burr-holes in a participating neurosurgical unit (figure 1). Time to surgery ranged from 0 to 44 days, with a median of 1 day and IQR of 1–3 days for the overall sample. Distribution of patients according to time to surgery is shown in table 1.

**Table 1:** Summary demographics of patients (n=656)

Measure	Statistic
Age (years, median, IQR)	78 (67–84)
Male (n, %)	449 (68)
Female (n, %)	207 (32)
Initial GCS (median, IQR)	14 (14–15)
Mild impairment (13–15, n, %)	540 (82)
Moderate impairment (9–12, n, %)	103 (16)
Severe impairment (3–8, n, %)	13 (2)
Comorbidities (n, %)	
Diabetes mellitus	109 (17)
Dementia	73 (11)
COPD	36 (5)
IHD	169 (26)
CVA	103 (16)
Malignancy	55 (8)
Anticoagulation (n, %)	
Warfarin use	124 (19)
Antiplatelet medication use	157 (24)
Initial mRS (median, IQR)	3 (2–4)
Length of stay (days, median, IQR)	7 (5–12)
Recollection / reoperation within 60 days (n, %)	59 (9)
Patients in each time to surgery (days) category (n, %)	
0 days	133 (20)
1 day	241 (37)
2 days	104 (16)
3–6 days	100 (15)
7+ days	78 (12)

COPD, Chronic obstuctive pulmonary disease; CVA, Cerebrovascular accident; GCS, Glasgow Coma Scale; IHD, Ischaemic heart disease; mRS, modified Rankin Scale.

**Figure 2:**
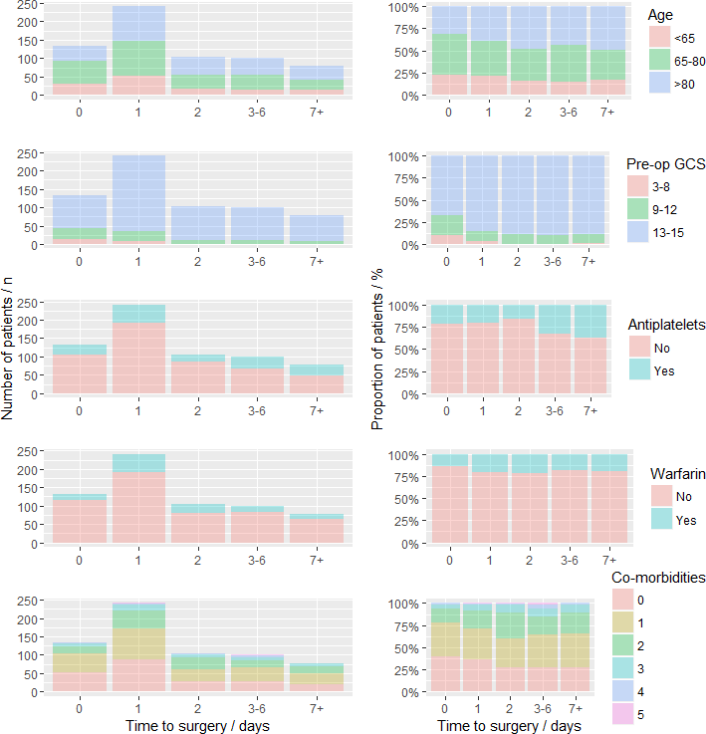
Distribution of patients by time to surgery, stratified by risk factor with absolute numbers and proportions by time category shown. GCS, Glasgow Coma Scale.

**Figure 3:**
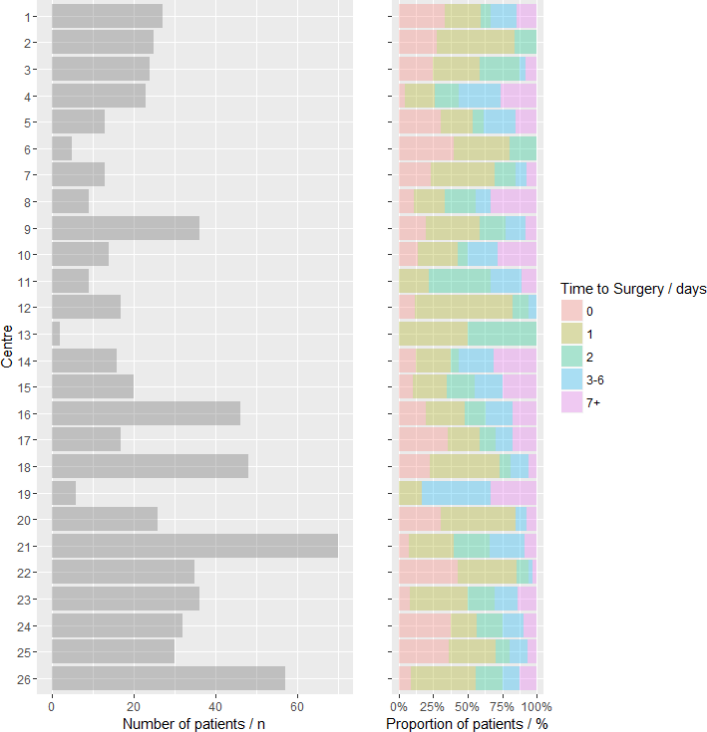
Distribution of patients by time to surgery stratified by neurosurgical center.

Descriptive statistics are presented in table 1 and figure 2. Of the patients with severe neurological impairment, defined by a preoperative GCS of <9, 11 out of 13 (85%) received burr-hole evacuation within 1 day following referral. Older patients were more likely to undergo an operation at a later stage, with a median age of 76 years for patients operated on the same day as the referral (ie, 0 days) compared with 80 years for patients operated at 7 or more days (p=0.019). A larger proportion of patients operated on 7 or more days after referral were taking antiplatelet medication compared with those operated on at 0 days (n=29/78, 38% vs n=29/133, 22%, p=0.017). This effect was less pronounced with warfarin, with a similar proportion of patients (n=18/133, 14% of patients) operated on 0 or 1 days versus 7 or more days (n=15/78, 19%; p=0.327). Distribution of patients across time to surgery categories differed between neurosurgical centers (figure 3). Patients were most commonly operated on between 0 and 2 days after referral in 21 neurosurgical centers. Three centers submitted cohorts with a median time from referral to surgery of 3 or more days.

### Factors influencing time to surgery

Mixed modelling results are shown in table 2. Time to surgery was modelled, based on a median value of 1 day, into ‘early’ if ≤1 day (n=374/656, 57.0%) or ‘late’ if ≥2 days (n=282/656, 43.0%). Other variables included age, initial mRS, preoperative GCS, use of antiplatelet medication, number of comorbidities and whether the CSDH was bilateral. The inclusion of the neurosurgical center significantly improved the model fit (AIC 867.8 as logistic regression vs 838.8 as generalized mixed model).

**Table 2:** Mixed model results of time to surgery

Variable	Estimate	SE	*Z*	Pr(>|z|)	OR (95% CI)
Age	0.019	0.007	2.661	0.008	1.02 (1.00 to 1.03)
Initial mRS	−0.178	0.084	−2.109	0.034	0.84 (0.71 to 0.99)
Preoperative GCS	0.172	0.056	3.067	0.002	1.19 (1.06 to 1.32)
Antiplatelet medications=yes	0.477	0.208	2.287	0.022	1.61 (1.07 to 2.42)
No of comorbidities	0.324	0.094	3.454	<0.001	1.38 (1.15 to 1.66)
CSDH=unilateral only	−0.445	0.204	−2.177	0.029	0.64 (0.43 to 0.96)

CSDH, chronic subdural hematoma; GCS, Glasgow Coma Scale; mRS, modified Rankin Scale.

The following variables were associated with a longer time to surgery: increasing age (OR 1.02 (95% CI 1.00 to 1.03), p=0.008), higher preoperative GCS (OR 1.19 (95% CI 1.06 to 1.32), p=0.002), higher number of comorbidities (OR 1.38 (95% CI 1.15 to 1.66), p<0.001), and the use of antiplatelet medications (OR 1.61 (95% CI 1.07 to 2.42), p=0.022). Conversely, a shorter time to surgery was associated with a higher admission mRS (OR 0.84 (95% CI 0.71 to 0.99), p=0.034) and the evacuation of a unilateral CSDH (OR 0.64 (95% CI 0.43 to 0.96), p=0.029).

Variables excluded from the model that were not significant and did not improve model fit included sex (p=0.527), preoperative use of warfarin (p=0.6757), and preoperative requirements of platelets (p=0.184), fresh frozen plasma (p=0.550), vitamin K (p=0.951), or clotting factors (p=0.170). Tested interaction terms (between age, initial mRS and comorbidities and between presence of bilateral CSDH and preoperative GCS) were not found to be significant.

### Impact of time to surgery on outcomes of interest

**Figure 4:**
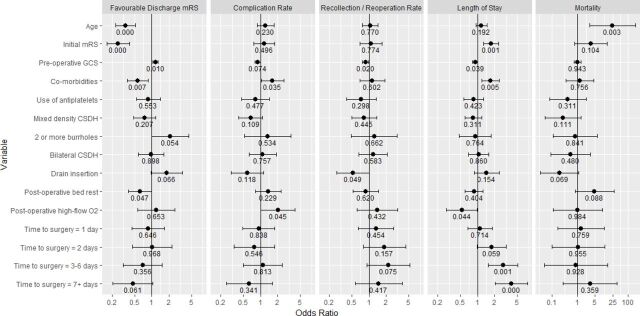
Multivariable logistic regression statistical results. Variables are shown with ORs, 95% CIs and p values labeled beneath each variable. Time to surgery is reported relative to an interval of 0 days. Discharge mRS was dichotomized into favorable as 1 (representing mRS of 0–3) or unfavorable of 0 (representing mRS of 4–6). Length of stay was dichotomized based on a median length of stay for the cohort of 7 days. mRS, modified Rankin Scale.

Logistic regression results across the five dependent variables of interest are shown in figure 4. Number of comorbidities was included alongside previously published variables in the multivariable model (see covariates for further details in the Methods section).

All comparisons of the various time intervals to surgery compared with 0 days were not found to be significantly related to a favorable outcome at discharge (p=0.061–0.837), rate of complications (n=88, p=0.428–0.957), rate of postoperative recollection requiring reoperation (n=59, p=0.075–0.454) or survival (n=11, p=0.367–0.948). Nevertheless, there was a trend indicating that a time interval of 7 days or more had lower odds of favorable outcome at discharge (p=0.061). Time to surgery was significantly associated with length of stay dichotomized based on a median of 7 days, with an increasing time to surgery associated with a greater length of stay (time to surgery 3–6 days OR 2.79 (95% CI 1.53 to 5.09), p=0.001, 7+ days OR 3.91 (95% CI 2.03 to 7.54), p<0.001, figure 4). Other variables significantly associated with length of stay included initial mRS (OR 1.88 (95% CI 1.39 to 2.55), p<0.001) and postoperative high-flow oxygen (OR 0.50 (95% CI 0.27 to 0.94), p=0.030).

## Discussion

Given the lack of definitive evidence available in the literature, there is uncertainty over the impact of the timing of surgery for CSDH on outcome. This observational cohort study, involving 656 patients with CSDH, investigated whether the time interval between referral and surgery impacted on functional outcome at discharge (mRS), complication rates, recurrence requiring reoperation, length of stay, and survival. While there was variation between units, the median time to surgery was 1 day, so in general it appears that most patients receive surgery in a timely manner.

Time to surgery demonstrated a positive linear relationship with length of stay. We found that a number of variables were associated with a longer time interval between referral and surgical intervention. These included increasing patient age, lower mRS, higher GCS, use of antiplatelet agents, presence of comorbidities, and presence of bilateral CSDH (p<0.05 for all variables).

Although interactions between age and comorbidities were tested and not found to be significant when building the model for this study, large epidemiological studies have demonstrated increasing comorbidities with age.^15 16^ Published reports by the National Confidential Enquiry into Patient Outcome and Death have highlighted the importance of multidisciplinary decision-making in surgery in the elderly where there is an increased risk of perioperative morbidity and mortality.^17^ Integrated care pathways have been effectively developed in Trauma and Orthopaedics for patients with a fractured neck of femur, with best practice tariffs if the patient is admitted under the joint care of a consultant orthopedic surgeon and a consultant geriatrician, and is postoperatively cared for by a geriatrician-directed multiprofessional rehabilitation team.^18^ Such pathways and systemic incentives have only recently started to be developed for elderly patients with CSDH, and seem to show early promising results including better preoperative optimization of patients.^19 20^

As antiplatelet agents are usually stopped prior to surgery, it is common practice to delay surgery for 5-10 days, depending on the agent, or transfuse platelets if surgery needs to be undertaken on an urgent basis.^21 22^ Notably, class III evidence demonstrates that waiting 3 days is sufficient to proceed to surgery after cessation of antiplatelet therapy with no recurrence after this time point.^23^

Evidence that time to surgery does not have a substantial adverse impact on clinical outcomes, as suggested in this study, could allay pressure from the managing team and encourage optimization for surgery. Our findings are in line with those of a recent retrospective cohort study from Sweden showing that increased time from CT scan to surgical evacuation for CSDH did not negatively impact outcomes, when surgery was performed within a clinically appropriate time frame.^24^ It appears that patients at risk of deterioration or those who are neurologically impaired are already being prioritized, as we found that a more unfavorable initial mRS, and lower GCS were associated with a shorter time to surgery. It would be ideal for the patient to undergo surgery as soon as practically feasible to begin the process of recovery and resolution of deficit(s), and we advocate for this to remain the ultimate aim in clinical practice. However, this has to be weighed against the importance of comprehensive preoperative assessment and optimization.

The trend between a time interval of 7 days or more and unfavorable outcome at discharge means that one cannot completely rule out a relationship between increasing time to surgery and worse functional outcome at discharge. Due to a paucity of studies in this field and risk of a type II error, further research should be carried out to investigate this potential relationship. We believe that decisions on when to intervene should always be guided by clinical assessment and the patient’s condition. When interpreting the results of this study, it must be remembered that this was an observational study without any randomization. Therefore, residual confounding and confounding by indication may still exist.

The linear relationship between time to surgery and length of stay has important service provision implications. The management of CSDH already represents a significant burden to neurosurgical service provision, with an estimated incidence of 8.2/100 000/year after 70 years of age.^25^ This is expected to increase in the context of increasing use of antiplatelet and anticoagulant therapy in an aging population.^26^ There are broader implications, with head-injured patients representing a significant source of financial deficits for healthcare organizations in the UK.^27^ Consequently, there is an important consideration to optimize neurosurgical service provision by reducing the length of stay for patients with CSDH. There is also limited evidence to suggest an increased length of stay is associated with a worse prognosis in CSDH.^28^

### Limitations and future work

Time to surgery was determined based on the time point of referral to neurosurgical services; a study looking at the time from the onset of neurological deterioration (eg, onset of hemiparesis) or time from diagnosis (CT scan) to surgery would be interesting, but these data were not available. Other pertinent data not available included the time of day the surgery was completed and whether or not the patient required a planned or unplanned admission to an intensive care unit. Discharge data also did not specify how long the patient remained in another hospital, such that overall in-hospital length of stay postoperatively could not be determined. Only patients undergoing burr-hole evacuation were included; however, this is the most common surgical procedure used to treat CSDH in the UK.^8 29^ It would be important to consider whether our findings are applicable to other patient groups such as those undergoing different procedures. Additionally, it would be important to establish whether risk stratification should be used when prioritizing patients for surgery.

Although this is a large sample of 656 patients, there is a risk of a type II error particularly given the conclusions derived from this study. This is exacerbated by selection bias due to the individual surgeon decision-making on optimal timing of surgery. The outcomes investigated were relatively short-term, with four out of five being assessed at discharge. The median length of stay in neurosurgical units was 7 days in the original study.^8^ The outcome with the longest follow-up time was recurrence rate, which was assessed for 60 days postoperatively; evidence suggests this is the highest risk time window and thereafter the risk of recurrence reduces.^30^ Conclusions about the longer-term outcome cannot be drawn at this stage. There is scope to expand this in further studies to lengthen follow-up to 6 months or longer. This would be helpful to understand the disease progression and impact on function,^31^ especially since previous studies highlighted that CSDH is comparable to hip fracture as a sentinel event for underlying systemic pathology with increased 1 year mortality.^31^ ^32^ This is particularly relevant for an elderly patient group; in this context, 6 month or 1 year outcomes are useful tools when completing holistic/comprehensive assessments. Additionally, complications following CSDH could be further classified into mild, moderate and severe groups based on clinical relevance, to ascertain if any of these groups are affected individually, as described in a recent population-based study investigating predictors of recurrence and complications following CSDH.^33^ This was not possible in this paper, as such data were not collected.

Despite its limitations, this is a large UK wide study including 656 patients treated across 26 neurosurgical centers. Therefore, it is likely to be representative of current UK neurosurgical practice. As previously stated, whether timeliness of surgical intervention in CSDH has an impact on patient outcome has not been studied widely, and uncertainty remains. This study adds to the existing knowledge and prompts a new set of questions. The findings of the study apply to a state-funded health system where universal health coverage is provided, and therefore the conclusions may not necessarily be generalizable to different healthcare systems.

Although our study did not evaluate a specific novel intervention, it is still useful to classify it according to the IDEAL (Idea, Development, Exploration, Assessment, Long-term study) framework.^34^ It can be viewed as an IDEAL stage 2b study, as exploratory analyses published as part of the primary paper^8^ identified a number of modifiable factors associated with better outcome, including use of two burr-holes and early mobilization. These are now being considered for evaluation with an IDEAL stage 3 study. Additionally, it can be viewed as an IDEAL stage 4 study with regards to the use of subdural drains, an intervention which was found to be beneficial in a previous high-quality randomized controlled trial.^35^ Our prospective, multicenter, observational study demonstrated that the national recurrence rate was 9%, very similar to that observed in the drain arm of the aforementioned trial. More importantly, the multivariate analysis showed that not using a drain independently predicted recurrence and unfavorable functional outcome, validating the effectiveness of subdural drains in a real-world setting.

## Conclusions

We have established that the current median time from referral to surgery is 1 day. The results do not demonstrate a relationship between time to surgery and clinical outcome following CSDH, although we cannot completely rule out a relationship between increasing time to surgery and worse outcome at discharge. In line with currently accepted best practice, all patients should be operated on in a timely manner and as soon as possible where there are clinical indications for surgery. Increasing time to surgery was associated with a longer length of stay; this has important service delivery implications. With this in mind, it is now important to conduct further research to (1) establish whether a pathway focusing on timely surgery with adequate preoperative optimization can improve outcomes, (2) implement longer follow-up times, and (3) validate our results.
